# The Elastic Behaviour of Sintered Metallic Fibre Networks: A Finite Element Study by Beam Theory

**DOI:** 10.1371/journal.pone.0143011

**Published:** 2015-11-16

**Authors:** Wolfram A. Bosbach

**Affiliations:** Engineering Department, University of Cambridge, Cambridge, United Kingdom; UC Santa Barbara, UNITED STATES

## Abstract

**Background:**

The finite element method has complimented research in the field of network mechanics in the past years in numerous studies about various materials. Numerical predictions and the planning efficiency of experimental procedures are two of the motivational aspects for these numerical studies. The widespread availability of high performance computing facilities has been the enabler for the simulation of sufficiently large systems.

**Objectives and Motivation:**

In the present study, finite element models were built for sintered, metallic fibre networks and validated by previously published experimental stiffness measurements. The validated models were the basis for predictions about so far unknown properties.

**Materials and Methods:**

The finite element models were built by transferring previously published skeletons of fibre networks into finite element models. Beam theory was applied as simplification method.

**Results and Conclusions:**

The obtained material stiffness isn’t a constant but rather a function of variables such as sample size and boundary conditions. Beam theory offers an efficient finite element method for the simulated fibre networks. The experimental results can be approximated by the simulated systems. Two worthwhile aspects for future work will be the influence of size and shape and the mechanical interaction with matrix materials.

## Introduction

### Background

The mechanics of metallic [1] and non-metallic [2] fibre networks have since long been the subject of research studies. One possible way to categorize the available studies is by the specific material which is investigated. Early and recent studies about cellulose material and paper can be found in [2–6]. Numerous studies exist about the mechanics of polymeric networks [7–11]. The modelling of polymeric non-woven fabrics and their particularly complex behaviour [12] has been in the focus of several research studies [13–17]. In the field of biomaterials, a big number of studies exists about the mechanics of actin networks and cytoskeletons [18–22]. A range of studies refers to theoretical networks whose geometries have been generated by computer code [23–31].

The mechanics of sintered, metallic fibre networks as used for the present study have been investigated by [1, 30–35]. In [36], an architectural characterization of the six network samples of the present study was published, together with experimentally obtained mechanical properties.

For finite element (FE) analyses of structures whose dimensions are dominated by their extension along only one axis, such as screws or fibres, beam theory can offer an efficient simulation method [37]. The foundations of beam theory were laid in [38–40] and are available in today’s textbooks [41]. For the application of boundary conditions (BC) to random fibre networks and for the determination of the representative volume element (RVE), [29, 42] have provided a much referred to concept [43–46]. By this concept, the RVE is obtained for a defined relative error for each physical property. Image acquisition by computed tomography (CT) scanning which uses the principle of Röntgen radiation [47] is a commonly used method for measuring the dimensions of metallic three-dimensional (3D) structures [48].

### Motivation and scope of the present study

The motivation of the present study was to predict previously unknown mechanical properties for metallic, sintered fibre networks. For that purpose, FE models were developed. The input geometries for the FE models were based on CT scans, acquired from real network samples in [36]. Experimental values for the network Young’s modulus from that same study were used for a validation of the FE models. The properties of these six network samples define the scope and at the same time also the limitations of the present study. Other metallic, sintered networks with similar but different properties will require a re-evaluation of the proposed FE models.

### Mathematical notation

Throughout the present study, the following notation is used: *x* for scalars, $\underline{x}$ for vectors, and $\underline{\underline{x}}$ for 2^*nd*^ rank tensors. Cube faces are written *X*. The vector product is given as “×” and dot product as “⋅”. Relations which are greater-than and approximately equal are written “≳”. If the relation is greater-than or equal “≥” is used.

## Materials and Methods

### Network samples and meshing step

**Fig 1:**
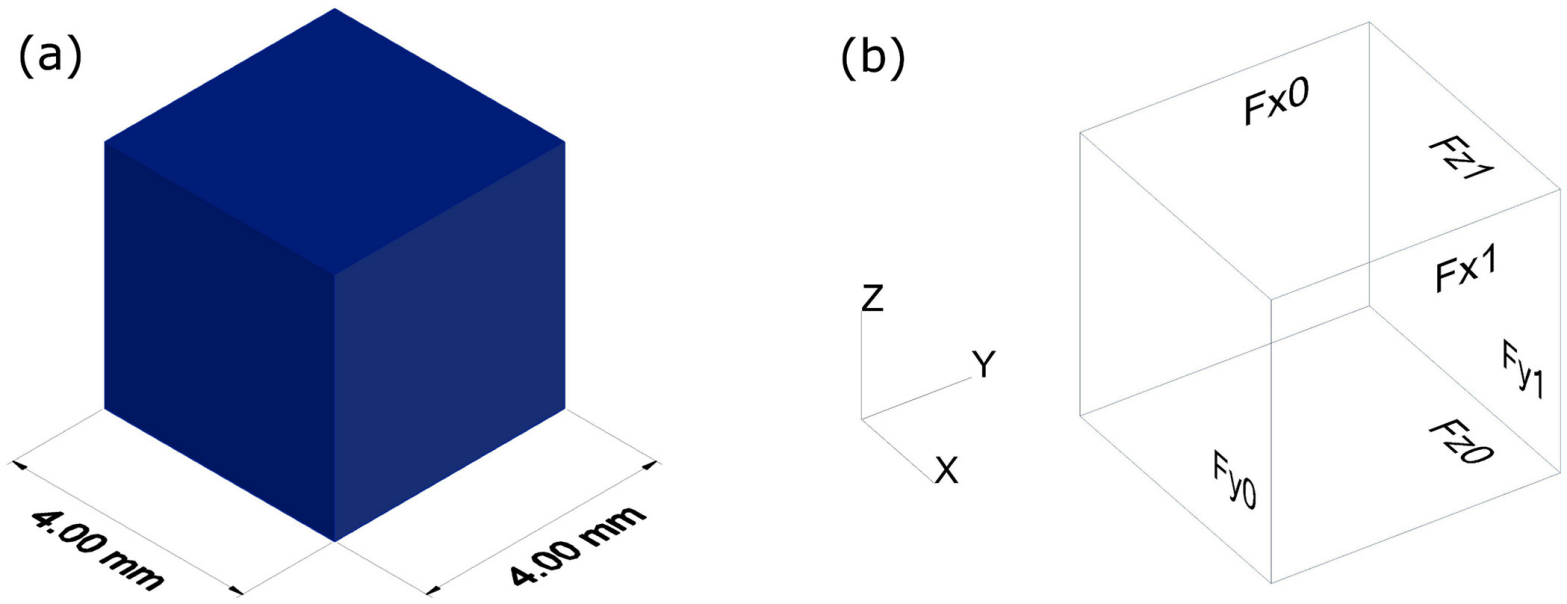
Sample cube dimensions and definition of cube faces. (a) Dimensions of fibre network cube *V* = 4^3^ mm^3^ (fibre network details in [36]) and (b) six quadratic cube faces (*F*_*x*0_, *F*_*x*1_, *F*_*y*0_, *F*_*y*1_, *F*_*z*0_, and *F*_*z*1_) forming *S*.

The present study uses as geometry input for the FE models the dimensions of six AISI (American Iron and Steel Institute) 316L network samples. The architectural network values have been published in [36] (see Table 1). Each scanned sample section is a cube of volume *V* = 4^3^ mm^3^ (see Fig 1a). Following the two-phase model in [29], *V* is split into fibre volume and void volume:

$$\begin{array}{c} V=V_{fibre}\cup V_{void} \end{array}$$ (1)

**Table 1:** AISI 316L network samples.

Sample	Fibre vol. frac.	Fibres	Fibre segments	Segment length	Beam elements
	*f* [%]	[-]	[-]	*λ* [μm]	[-]
316L-10%-No.1	10	2,891	22,913	235	700,515
316L-10%-No.2	10	2,815	22,789	239	709,427
316L-15%-No.1	15	4,326	37,049	191	929,249
316L-15%-No.2	15	4,920	41,479	181	1,029,453
316L-20%-No.1	20	6,138	59,936	153	1,265,910
316L-20%-No.2	20	6,400	59,949	153	1,269,925

Complete documentation of geometries in [36].

Equally, the volume boundary ∂*V* is defined to consist of a section of fibre boundary and a section of void boundary on the cube surface *S*:

$$\begin{array}{c} \partial V=\partial V_{fibre}\cup \partial V_{void} \end{array}$$ (2)

Six quadratic cube faces form *S* (see Fig 1b), two of them perpendicular each to one of the three axes:

$$\begin{array}{c} S=F_{x0}\cup F_{y0}\cup F_{z0}\cup F_{x1}\cup F_{y1}\cup F_{z1} \end{array}$$ (3)

**Fig 2:**
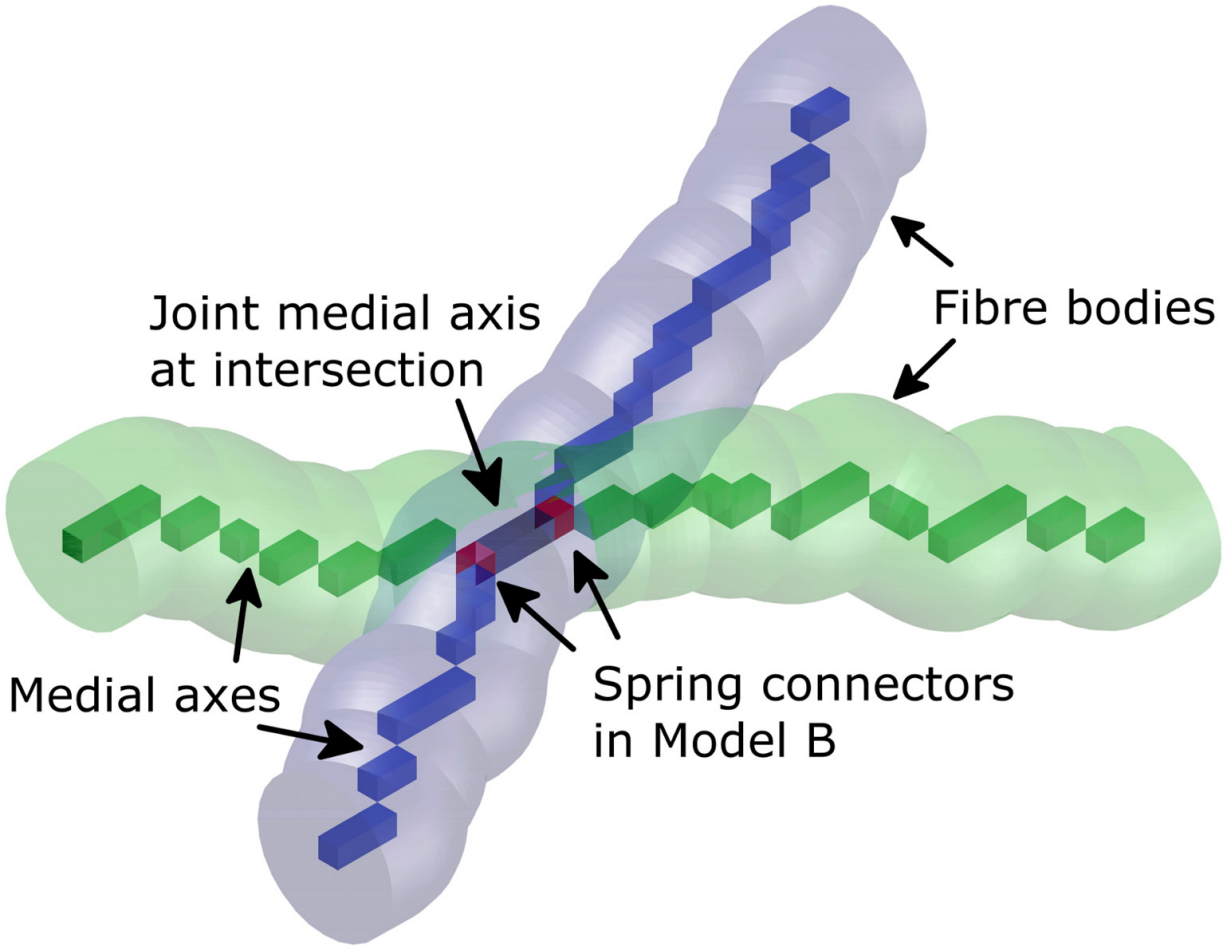
Medial axis model. Sketch of two intersecting fibre bodies with corresponding medial axes (green and blue) and joint medial axis at intersection (black) obtained by skeletonisation algorithm [50–52] in [36] and positions of spring connectors in Model B of the present study (red).

Two samples each are manufactured with a fibre volume fraction *f* of 10, 15, and 20%. In [36], the fibre production process by bundle drawing [49], the network manufacturing at N.V. Bekaert S.A. (Belgium), the CT scan acquisition at General Electric (resolution 7.75 μm), and the applied skeletonisation algorithm [50–52] are discussed in detail. The skeletonisation algorithm reduces the 3D fibre bodies to their medial axes which are strings of voxels (see Fig 2). The medial axis models obtained for [36] are transferred in the present study into beam assemblies and run as FE models. The approximately hexagonal fibre cross-section is simulated as a round cross-section of radius *R* = 20 μm.

### FE models

For the present study, two FE models are implemented (see Table 2). Model A and Model B are based on five specifications:

1. Linear elasticity and static equilibrium,
2. Euler-Bernoulli or Timoshenko beam elements as AISI 316L fibres,
3. Rigid joints or torsional springs as inter-fibre joint models at contact points between fibre paths (see Fig 2),
4. BC as defined in Eqs (8) and (11), and
5. the new introduced model parameter of BC-depth *h*_*BC*_.

**Table 2:** FE models.

	Joint model	Scaling factor	Beam element	BC-type	BC-depth
**Model A**	Rigid joint	*- not applicable -*	B31, B32, B33	KUBC, MBC	0 μm ≤ *h*_*BC*_ ≤ 155.00 μm
**Model B**	Spring joint	*s* ∈ {5, 10, 30, 300, 3000} μm	B31, B32	KUBC, MBC	*h*_*BC*_ = 77.50 μm = const

#### Linear elasticity and static equilibrium conditions

For mechanical FE analyses, the simulated system is transferred into a representation by the global stiffness matrix $\underline{\underline{K}}$; the most common assembly method being the *direct stiffness method* [53]. $\underline{\underline{K}}$ links the global load vector $\underline{F}$ and the global displacement vector $\underline{u}$:

$$\begin{array}{c} \underline{F}=\underline{\underline{K}}\;\cdot \;\underline{u} \end{array}$$ (4)

The meshed geometries and material specifications define the variables of $\underline{\underline{K}}$. In the present study, the material is assumed to behave linearly elastic (i.e. fulfil Hook’s law that stress and strain are linked by the Young’s modulus: *σ* = *Eϵ* [54]). In this case, the variables of $\underline{\underline{K}}$ become constants. The AISI 316L material stiffness is simulated as *E*_*fibre*_ = 200GPa with a Poisson’s ratio *ν* = 0.3 which expresses the material’s lateral contraction as fraction of the axial extension. For plastic or non-elastic simulations, the variables of $\underline{\underline{K}}$ are functions of e.g. force or displacement.

BC are imposed in FE by prescribed values for entries of $\underline{F}$ and $\underline{u}$. The non-prescribed entries of $\underline{F}$ and $\underline{u}$ are determined by the FE solver (present study: Abaqus 6.13 [55]) with solutions respecting the equilibrium conditions of forces in Eq (5) and of moments in Eq (6) [56]. Both equations adopt the Lagrangian reference frame [57]. (A comparison to the Eulerian reference frame and its advantages for the modelling of fluids is available in [58].)

$$\begin{array}{c} \int_{S}\underline{t}\;\;dS+\int_{V}\underline{f}\;\;dV=0\;\;\;\;\;\left(\text{with}\;\;\underline{t}=\underline{\underline{\sigma }}\;\cdot \;{\underline{n}}^{out}\right) \end{array}$$ (5)

$$\begin{array}{c} \int_{S}\left(\underline{x}\times \underline{t}\right)\;\;dS+\int_{V}\left(\underline{x}\times \underline{f}\right)\;\;dV=0 \end{array}$$ (6)

The surface traction vector $\underline{t}$ is obtained as the product of the Cauchy stress tensor $\underline{\underline{\sigma }}$ and the unit outward normal ${\underline{n}}^{out}$. (A detailed discussion of $\underline{\underline{\sigma }}$ is available in today’s textbooks [59].) In the mechanical simulations of the present study, body force per volume $\underline{f}$ is neglected; gravitational forces or magnetic forces being typical examples for $\underline{f}$. The point vector $\underline{x}$ specifies the location of a point relative to the origin; i.e. also the equilibrium of moments in Eq (6) being taken about the origin.

#### Beam elements

One Euler-Bernoulli beam element (B33) and two Timoshenko beam elements, one with linear interpolation (B31) and one with quadratic interpolation (B32), are implemented for the present study. Table 3 contains the complete list of implemented Abaqus elements. The neglected shear strain of the Euler-Bernoulli beam and the advanced Timoshenko beam are further discussed in [41] for the simplified 2D case. Literature recommends in general for beam assemblies which include short beam elements the Timoshenko beam [28, 60–62]. For this case, [62] has documented an overestimation of the structural stiffness by Euler-Bernoulli beam elements.

**Table 3:** Implemented Abaqus elements.

Element Type	Abaqus identifier	Interpolation/Connection
Timoshenko Beam	B31	3D Linear
	B32	3D Quadratic
Euler-Bernoulli Beam	B33	3D Cubic
Spring connector	CONN3D2	3D Join & torsional spring

Complete software documentation in [55].

#### Inter-fibre joint models

Model A is simulated for rigid inter-fibre joints. Torsional spring elements (CONN3D2) are inserted into the medial axis models in Model B between intersecting fibre paths (see Fig 2). This concept for adaptive joint strength of Model B is proposed for comparable cases in publications such as [63]. In the present study, the inter-fibre joint stiffness *K*_*joint*_ is defined:

$$\begin{array}{c} K_{joint}=sE_{fibre}A_{fibre} \end{array}$$ (7)

The scaling factor *s* [m] in Eq (7) allows the directed variation of *K*_*joint*_ for an approximation of the experimental values of [36]. The cross-sectional area *A*_*fibre*_ and *E*_*fibre*_ are included as they relate the modelled value of *K*_*joint*_ to the fibre dimensions and to the fibre stiffness. Alternative model variables could be chosen too.

#### Kinematic uniform BC and load cases

In [42], a simulation set of kinematic uniform BC (KUBC), static uniform BC (SUBC), and periodic BC (PBC) was proposed for a random heterogeneous composite. KUBC are applied in the present study as defined in Eq (8). In the case of KUBC, a macroscopic strain tensor $\underline{\underline{E}}$ imposes the displacement vector $\underline{u}$ on all $\underline{x}$ located on ∂*V*:

$$\begin{array}{c} \underline{u}=\underline{\underline{E}}\;\cdot \;\underline{x}\;\;\;\forall \;\underline{x}\in \partial V \end{array}$$ (8)

Six independent load cases are implemented in the present study by KUBC for the network samples: *i* = 1 to 3 for the simulation of tensile tests along the axes x, y, z and *i* = 4 to 6 for the corresponding shear tests. The combination of all six load cases leads to the symmetrical stiffness matrix $\underline{\underline{C}}$, where the six entries *C*_*ii*_ on the main diagonal stand for the Young’s moduli *E* and Shear moduli *G* [64, 65]:

$$\begin{array}{cc} \left\{ \begin{array}{c} \sigma_{x}\\ \sigma_{y}\\ \sigma_{z}\\ \tau_{yz}\\ \tau_{zx}\\ \tau_{xy} \end{array}\}=\right. & \left[\begin{array}{cccccc} C_{11} & C_{12} & C_{13} & C_{14} & C_{15} & C_{16}\\ • & C_{22} & C_{23} & C_{24} & C_{25} & C_{26}\\ • & • & C_{33} & C_{34} & C_{35} & C_{36}\\ • & • & • & C_{44} & C_{45} & C_{46}\\ • & • & • & • & C_{55} & C_{56}\\ • & • & • & • & • & C_{66} \end{array}]\{\begin{array}{c} \epsilon_{x}\\ \epsilon_{y}\\ \epsilon_{z}\\ \gamma_{yz}\\ \gamma_{zx}\\ \gamma_{xy} \end{array}\right\}\\ \text{with}:\;\; & C_{11}=E_{x},C_{22}=E_{y},C_{33}=E_{z}\\ & C_{44}=G_{yz},C_{55}=G_{zx},C_{66}=G_{xy} \end{array}$$ (9)

The general 4^*th*^ rank stiffness tensor contains in total 81 components. The simplification to only 21 independent variables in $\underline{\underline{C}}$ is achieved by energy considerations and symmetry [66].

#### Mixed BC

The application of further BC to random fibre networks requires additional considerations [29]. In the case of SUBC, a macroscopic traction vector $\underline{\underline{\Gamma }}$ is imposed on ∂*V*. PBC add a periodic fluctuation term $\underline{v}$. In [29], it is discussed for random fibre networks that considering:

$$\begin{array}{c} \underline{\underline{\sigma }}=0\;\;\forall \;\underline{x}\in V_{void} \end{array}$$ (10)

$\underline{\underline{\Gamma }}$ can’t be prescribed on ∂*V*_*void*_, only on ∂*V*_*fibre*_. Due to the randomness of the fibre network geometry, the equilibrium conditions in Eqs (5) and (6) are not fulfilled when $\underline{\underline{\Gamma }}$ is imposed. For overcoming this, mixed BC (MBC) are proposed by [29] and adopted in the present study for the simulation of tensile testing along the axes x and y by one independent load case each (load case *i* = 1 and 2, definition see above). A macroscopic strain tensor ${\underline{\underline{E}}}^{prim}$ imposes the primary displacement ${\underline{u}}^{prim}$ along the axis of tension only:

$$\begin{array}{c} {\underline{u}}^{prim}={\underline{\underline{E}}}^{prim}\;\cdot \;\underline{x}\;\;\;\forall \;\underline{x}\in \{\begin{array}{c} F_{x0}\cup F_{x1},\;\text{for}\;i=1\;\;\text{(tensile-x)}\\ F_{y0}\cup F_{y1},\;\text{for}\;i=2\;\;\text{(tensile-y)} \end{array} \end{array}$$ (11)

Along the two non-prescribed axes, a secondary displacement ${\underline{u}}^{sec}$ is caused as a consequence of the imposed ${\underline{u}}^{prim}$. The total deformation of the sample is obtained under MBC as: ${\underline{u}}^{total}={\underline{u}}^{prim}+{\underline{u}}^{sec}$.

The results obtained in [29] for the elastic modulus of computer generated random fibre networks without preferred orientation direction predict greater stiffness for KUBC: *C*_MBC_ < *C*_KUBC_.

#### BC-depth

**Fig 3:**
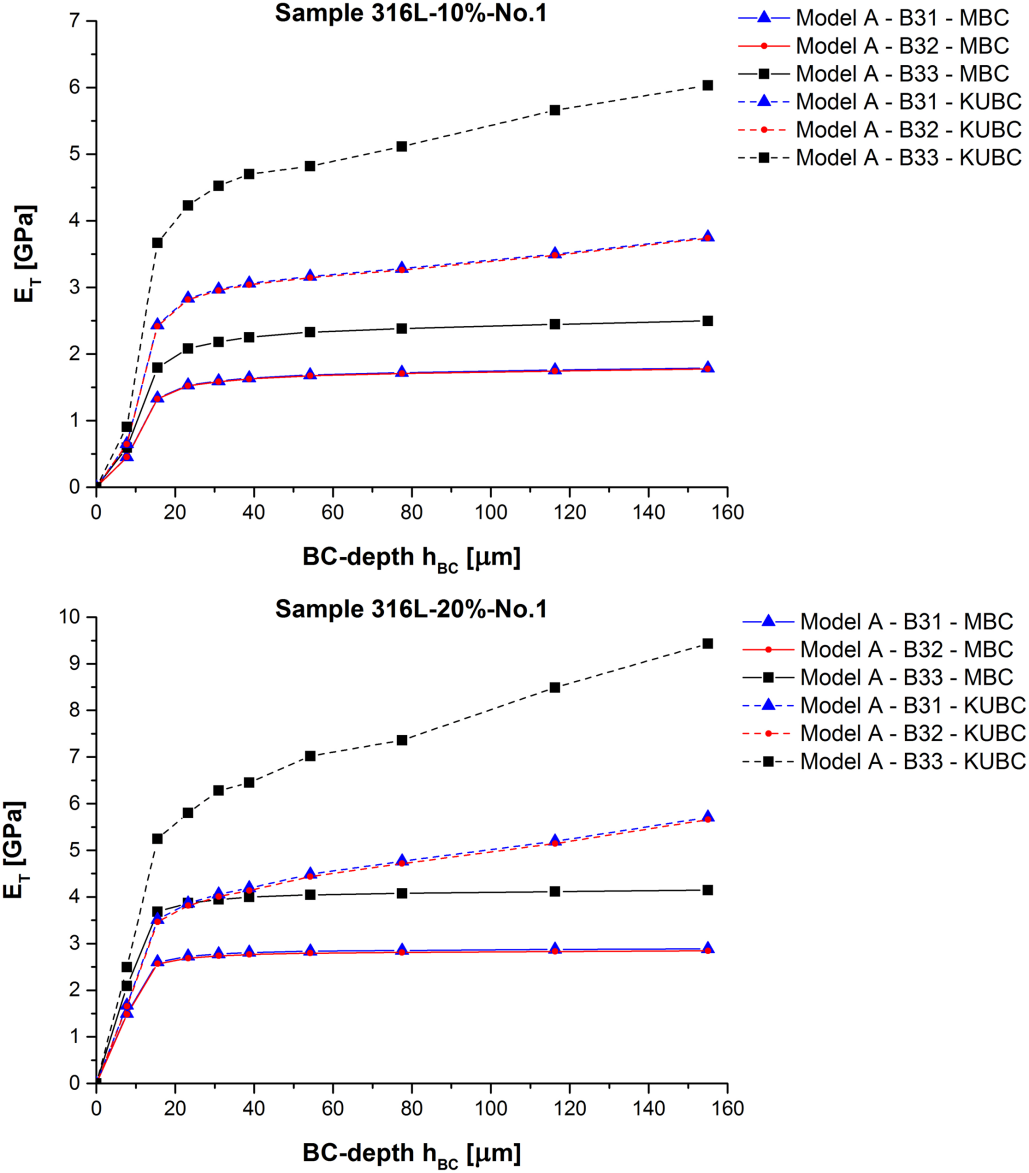
Model A—Influence of BC-depth. Obtained *E*_*T*_ values of samples 316L-10%-No.1, and 316L-20%-No.1 depending on *h*_*BC*_.

**Fig 4:**
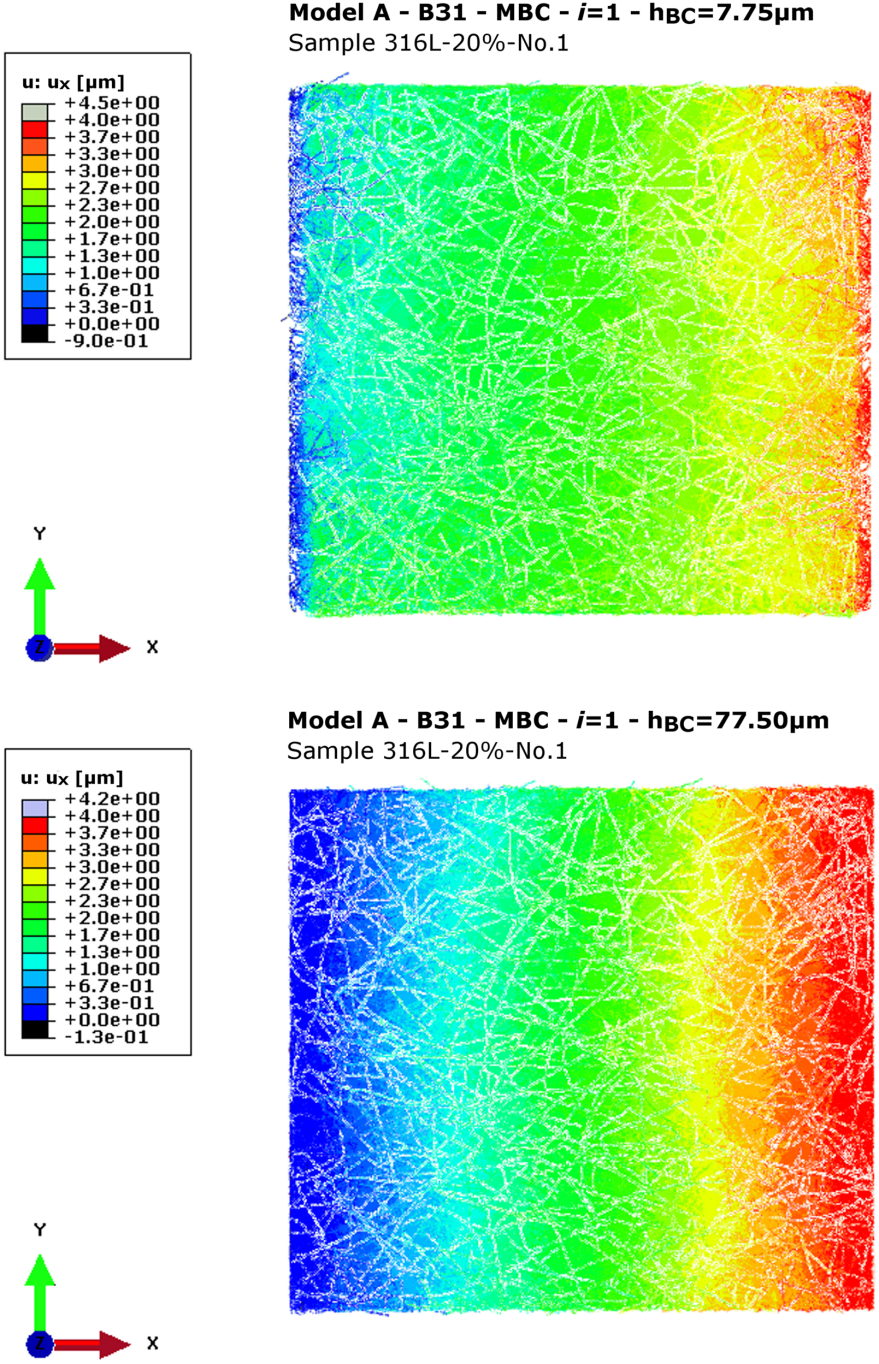
Model A—Deformation plot. Obtained *u*_*x*_ [μm] for sample 316L-20%-No.1 with *h*_*BC*_ = 7.75 or 77.50 μm in load case *i* = 1 (tensile-x).

The present study extends the BC model of Eqs (8) and (11) for the investigated material by the variable of *h*_*BC*_. The value of *h*_*BC*_ prescribes to which depth into the material along the inward normal ${\underline{n}}^{in}$ BC are imposed on ∂*V*. The reason for the model modification is the obtained structural response which will be discussed further in the following section by Model A in Figs 3 and 4.

### Representative volume element determination

For the determination of the RVE size *V*_RVE_, the present study relied on an algorithm proposed by [42]. This algorithm has been applied since its publication to random fibre networks in [29, 32] and other materials [43–46].

In [42], *V*_RVE_ is obtained as a probability estimate. The RVE is understood as the minimum cube volume *V*_RVE_ for which a particular physical property *Z* after *n* realisations can be determined within the margin of the relative error *ϵ*_*rel*_. Due to this definition, *V*_RVE_ is in each case a function of these three variables (see Eq (20)).

In order to acquire the input for the RVE algorithm, the available material sample is split into sets of sub-samples of *V*_*k*_ (with $L_{k}=V_{k}^{1/3}$) of *n*_*k*_ realisations. The size function of the RVE is then determined by a regression analysis for the standard deviation *D*_*Z*(*V*_*k*_)_ of *Z* over *V*_*k*_. In [45], the mean for *V*_*k*_ of a physical property *Z*_(*V*_*k*_)_ and the respective variation $D_{Z(V_{k})}^{2}$ are calculated as follows:

$$\begin{array}{c} Z_{\left(V_{k}\right)}=\frac{1}{n_{k}}\sum_{j=1}^{j=n_{k}}Z_{\left(V_{k},j\right)} \end{array}$$ (12)

$$\begin{array}{c} D_{Z(V_{k})}^{2}=\frac{1}{n_{k}-1}\sum_{j=1}^{j=n_{k}}{\left(Z_{\left(V_{k},j\right)}-\bar{Z_{\left(V_{k}\right)}}\right)}^{2} \end{array}$$ (13)

Using the expressions gained in Eqs (12) and (13), the absolute sampling error *ϵ*_*abs*_ and *ϵ*_*rel*_ are defined for this algorithm, following [29, 42, 67]:

$$\begin{array}{c} \epsilon_{abs}=\frac{2D_{Z(V)}}{\sqrt{n}} \end{array}$$ (14)

$$\begin{array}{c} \epsilon_{rel}=\frac{\epsilon_{abs}}{Z} \end{array}$$ (15)

*V* is linked to the variation $D_{Z(V)}^{2}$ and the point variance $D_{Z}^{2}$ [29, 42, 68]:

$$\begin{array}{c} D_{Z(V)}^{2}=D_{Z}^{2}\times {\left(\frac{A_{3}}{V}\right)}^{\alpha } \end{array}$$ (16)

$$\begin{array}{c} D_{Z}^{2}=f\left(1-f\right){\left(Z_{fibre}-Z_{void}\right)}^{2} \end{array}$$ (17)

*Z*_*fibre*_ takes in the present study the value of the respective physical property in *V*_*fibre*_, *Z*_*void*_ of the property in *V*_*void*_ (with *E*_*void*_ = 0). Eq (16) requires from the sample volume *V* [29]:

$$\begin{array}{c} V\gg A_{3} \end{array}$$ (18)

The term integral range *A*_3_ [69–72] provides a mathematical measure for the characterisation of random structures.

Inserting Eq (14) into Eq (16) yields Eq (19) which provides for *Z* the required relation between *V*, *ϵ*_*abs*_, and *n* [29, 42]:

$$\begin{array}{c} n=\frac{4}{\epsilon_{abs}^{2}}D_{Z}^{2}{\left(\frac{A_{3}}{V}\right)}^{\alpha } \end{array}$$ (19)

Eq (19) can be transformed into the final RVE-formula. Now, *V* stands for the sought variable *V*_RVE_ [29, 42]:

$$\begin{array}{c} V_{\text{RVE}}={\left(\frac{4}{n\;\epsilon_{abs}^{2}}D_{Z}^{2}A_{3}^{\alpha }\right)}^{\frac{1}{\alpha }}={\left(\frac{4}{n\;\epsilon_{rel}^{2}{\bar{Z}}^{2}}D_{Z}^{2}A_{3}^{\alpha }\right)}^{\frac{1}{\alpha }} \end{array}$$ (20)

In Eq (20), only two unknown variables remain whose values need to be determined for the calculation of *V*_RVE_, i.e. *A*_3_ and the exponential RVE-factor *α*. Linear regression (*y* = *ax* + *b*) of the log-transformed relation given in Eq (16) produces these two values [29, 42]:

$$\begin{array}{ccc} log(D_{Z(V)}^{2}) & = & -\alpha \;log\left(V\right)+(log\left(D_{Z}^{2}\right)+\alpha \;log\left(A_{3}\right))\\ y & := & \;log(D_{Z}^{2}\left(V\right))\\ a & := & \;-\alpha \\ x & := & \;log(V)\\ b & := & \;log\left(D_{Z}^{2}\right)+\alpha \;log\left(A_{3}\right) \end{array}$$ (21)

## Results and Discussion

### FE modelling of experimental in-plane Young’s modulus

The transverse Young’s modulus *E*_*T*_ ($=\frac{1}{2}\left(E_{x}+E_{y}\right)$) is obtained through the in-plane load cases *i* = 1 and 2 by Model A and B. The experimental results of [36] are approximated by Model B.

#### Model A—Rigid joint model

The value of *h*_*BC*_ has a non-negligible influence on the obtained *E*_*T*_ in particular for *h*_*BC*_ < 77.50 μm (see Fig 3 for the exemplary values of sample 316L-10%-No.1, and 316L-20%-No.1). Whether *h*_*BC*_ still changes *E*_*T*_ for *h*_*BC*_ ≥ 77.50 μm depends on the imposed BC type. The deformation plots demonstrate for MBC that for low *h*_*BC*_ the applied BC cause a mechanical response almost exclusively in the close proximity to the two constrained cube faces *F*_*x*0_ and *F*_*x*1_ (see Fig 4). For greater *h*_*BC*_, a nearly linear displacement increase through the sample along the axis of tension can be observed in analogy to the increased *E*_*T*_ in Fig 3.

The change from linear to quadratic interpolation decreases the *E*_*T*_ obtained by Timoshenko beams only marginally (see Tables 4 and 5). *E*_*T*_ increases considerably when Euler-Bernoulli beams are simulated:

$$\begin{array}{c} E_{T,B32}≲E_{T,B31}<E_{T,B33} \end{array}$$ (22)

**Table 4:** Model A—Influence of beam type on *E*_*T*_ under MBC.

BC: MBC		*h*_*BC*_:				
316L-Sample:	Ratio:	7.75	38.75	77.50	155.00	[μm]
10%-No.1	*E*_*T*,B33_/*E*_*T*,B31_	131.05%	137.45%	138.44%	139.62%	[-]
15%-No.1	*E*_*T*,B33_/*E*_*T*,B31_	137.63%	143.10%	143.88%	148.88%	
20%-No.1	*E*_*T*,B33_/*E*_*T*,B31_	139.45%	142.35%	142.79%	143.53%	
10%-No.1	*E*_*T*,B32_/*E*_*T*,B31_	99.44%	99.31%	99.26%	99.27%	[-]
15%-No.1	*E*_*T*,B32_/*E*_*T*,B31_	99.12%	99.00%	98.99%	98.99%	
20%-No.1	*E*_*T*,B32_/*E*_*T*,B31_	98.65%	98.49%	98.47%	98.47%	

**Table 5:** Model A—Influence of beam type on *E*_*T*_ under KUBC.

BC: KUBC		*h*_*BC*_:				
316L-Sample:	Ratio:	7.75	38.75	77.50	155.00	[μm]
10%-No.1	*E*_*T*,B33_/*E*_*T*,B31_	139.02%	153.55%	155.84%	160.58%	[-]
15%-No.1	*E*_*T*,B33_/*E*_*T*,B31_	147.34%	151.64%	161.01%	168.81%	
20%-No.1	*E*_*T*,B33_/*E*_*T*,B31_	148.79%	153.94%	154.38%	165.32%	
10%-No.1	*E*_*T*,B32_/*E*_*T*,B31_	99.34%	99.35%	99.39%	99.50%	[-]
15%-No.1	*E*_*T*,B32_/*E*_*T*,B31_	99.04%	99.11%	99.21%	99.34%	
20%-No.1	*E*_*T*,B32_/*E*_*T*,B31_	98.63%	98.77%	98.91%	99.12%	

The reduced number of prescribed degrees of freedom (DOF) in the case of MBC reduces also the value of *E*_*T*_ when compared to KUBC:

$$\begin{array}{c} E_{T,\text{MBC}}<E_{T,\text{KUBC}} \end{array}$$ (23)

The findings in Eqs (22) and (23) hold true irrespectively of the *h*_*BC*_ value and confirm the prediction of [62] and [29] respectively.

#### Model B—Spring joint model

**Fig 5:**
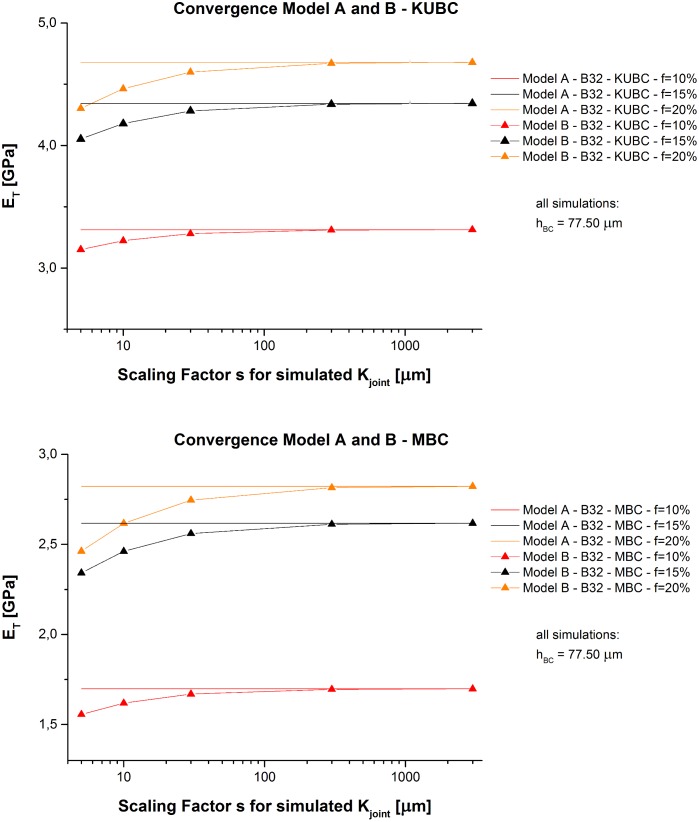
Model A and B—Convergence of results. Obtained *E*_*T*_ values of Model A and Model B depending on *s*.

*E*_*T*_ is obtained by Model B for values of *s* defined in Table 2 with *h*_*BC*_ = 77.50 μm. This *h*_*BC*_ value is chosen because of the obtained *E*_*T*_ magnitude under MBC (similar to the experimental values of [36]) and because of the considerably reduced *dE*_*T*_/*dh*_*BC*_ at this value. As required, results for Model B tend towards Model A for greater *s* independently of the applied BC (see Fig 5):

$$\begin{array}{c} \lim_{s[m]\to \infty }E_{T,B(s)}=E_{T,A} \end{array}$$ (24)

**Fig 6:**
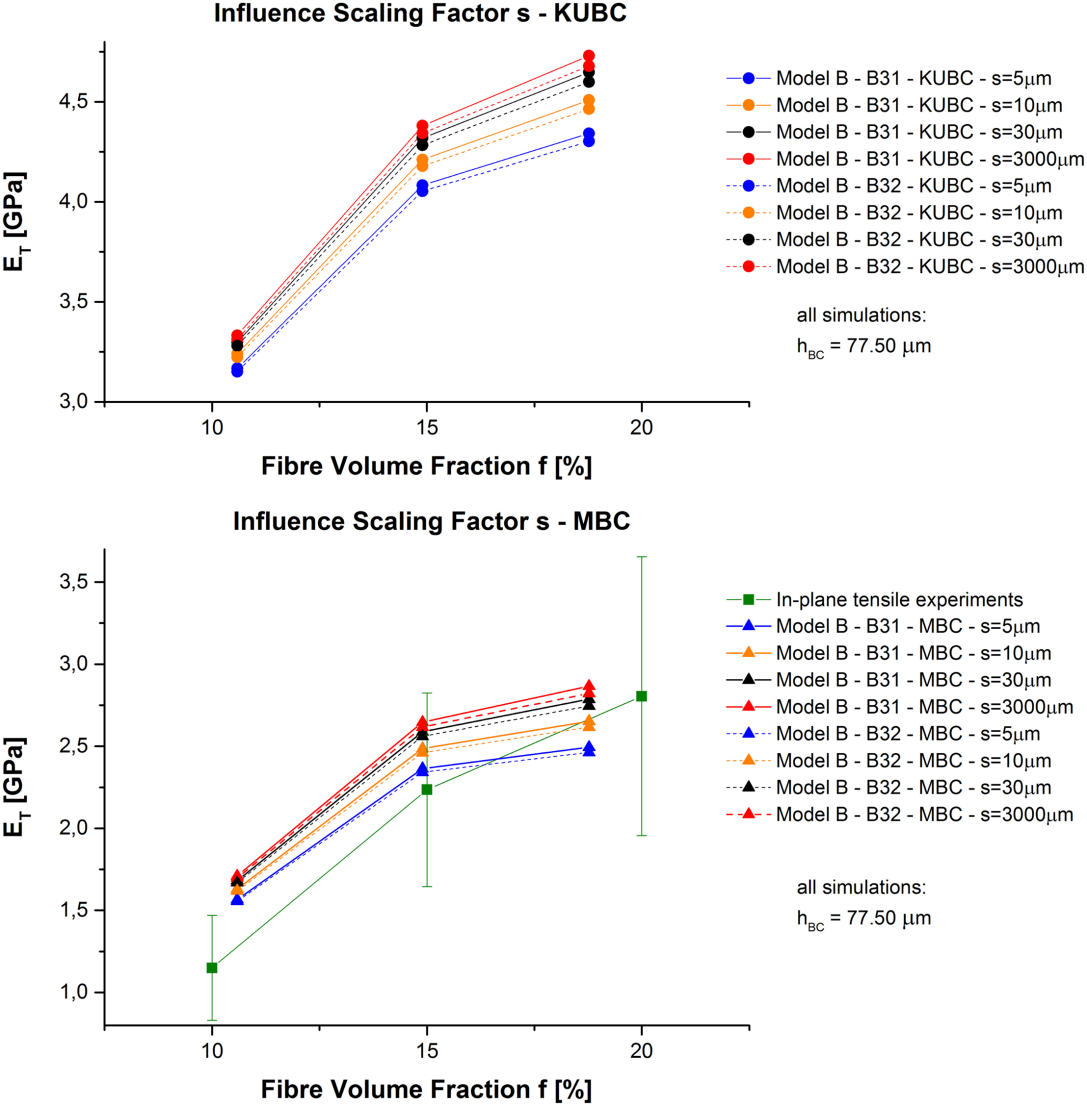
Model B—Comparison experiments and simulation. Obtained *E*_*T*_ values of Model B depending on *f* and experimental in-plane tensile testing results of [36].

Under MBC with only two constrained cube faces, *E*_*T*_ results match better the experimentally obtained stiffness values of in-plane tensile testing of [36]. The best match of Model B under MBC is obtained for *s* = 5 μm (see Fig 6) which is used for the following analyses.

### Stiffness matrix and transverse isotropy

**Fig 7:**
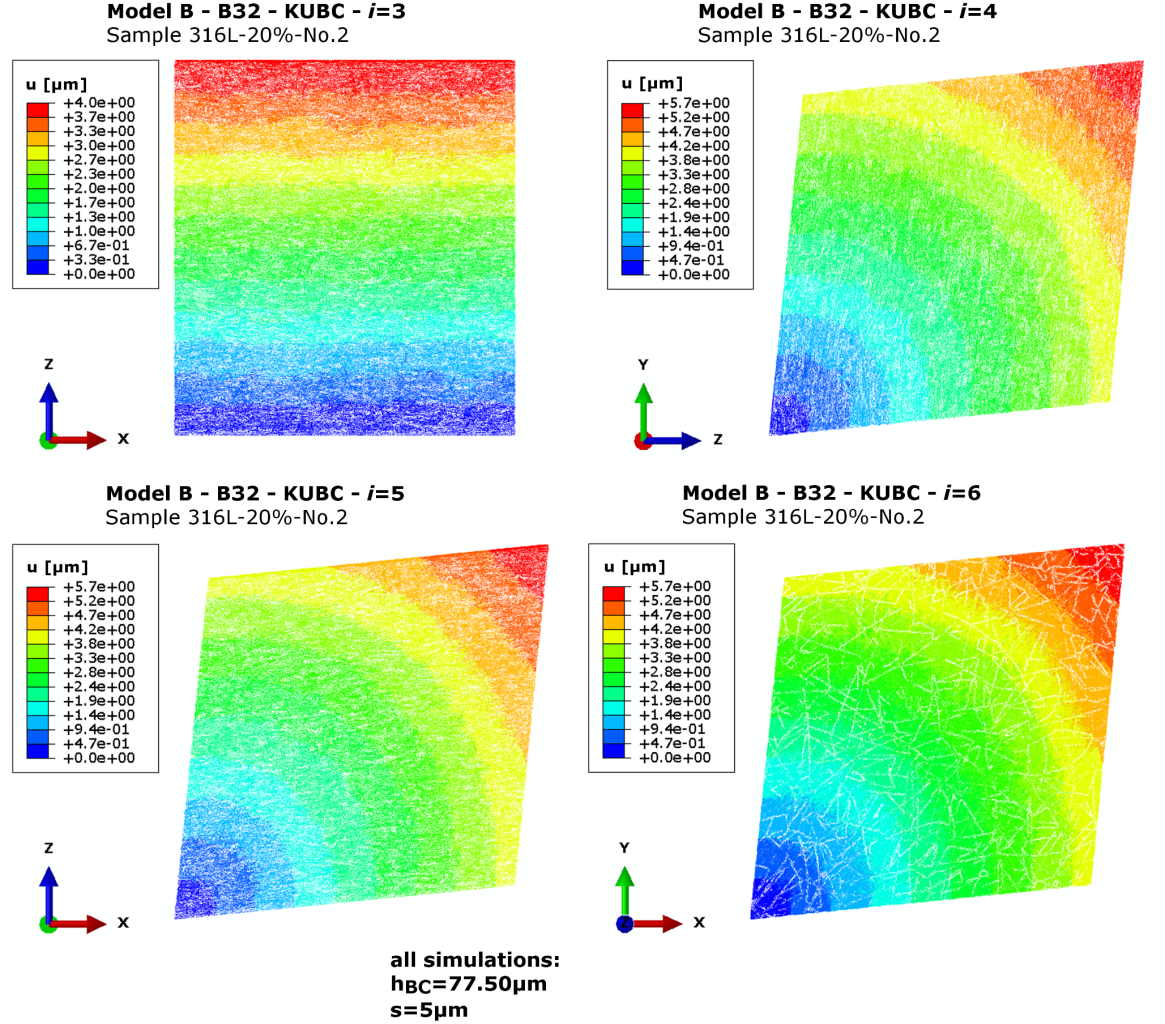
Model B—Deformation plot. Obtained *u* [μm] for sample 316L-20%-No.2 in load cases *i* = 3 to 6.

The dominantly in-plane fibre orientation of the network samples (see [36, 73] for detailed analyses) influences the mechanical behaviour to a great extent when the remaining load cases *i* = 3 to 6 (see Fig 7 for sample 316L-20%-No.2) are obtained. It can be described as transversely isotropic.

Eqs (25)–(27) present the elastic moduli together with the confidence intervals [*C*_*ij*_ ± 2*D*_*C*_*ij*__] under KUBC for B32 of each *f*. All non-bold entries would be expected to equal zero. However, they only nearly vanish which is caused by the numerical artefacts. *C*_11_ deviates from *C*_22_ by a maximum error of 6.13% for *f* = 10%. The conditions that *C*_44_ ≈ *C*_55_ and *C*_13_ ≈ *C*_23_ show the biggest errors for *f* = 15% (5.67% and 4.80% respectively). The obtained error for $C_{66}\approx \frac{1}{2}\left(C_{11}-C_{12}\right)$ varies between 3.20% for *f* = 10% and 11.27% for *f* = 20%.

$$\begin{array}{c} {\bar{\left[C\right]}}_{f=10\%}^{B32}=[\begin{array}{cccccc} 3.25\pm 0.27 & 1.01\pm 0.06 & 0.10\pm 0.03 & 0.02\pm 0.03 & 0.06\pm 0.04 & 0.07\pm 0.08\\ \cdot & 3.05\pm 0.09 & 0.10\pm 0.03 & 0.06\pm 0.08 & 0.03\pm 0.02 & 0.12\pm 0.08\\ \cdot & \cdot & 0.32\pm 0.01 & 0.02\pm 0.02 & 0.02\pm 0.03 & 0.02\pm 0.02\\ \cdot & \cdot & \cdot & 0.17\pm 0.02 & 0.01\pm 0.00 & 0.04\pm 0.04\\ \cdot & \cdot & \cdot & \cdot & 0.17\pm 0.02 & 0.02\pm 0.02\\ \cdot & \cdot & \cdot & \cdot & \cdot & 1.09\pm 0.04 \end{array}]\text{GPa} \end{array}$$ (25)

$$\begin{array}{c} {\bar{\left[C\right]}}_{f=15\%}^{B32}=[\begin{array}{cccccc} 4.06\pm 0.07 & 1.28\pm 0.10 & 0.18\pm 0.06 & 0.04\pm 0.05 & 0.13\pm 0.21 & 0.11\pm 0.05\\ \cdot & 4.06\pm 0.10 & 0.17\pm 0.03 & 0.11\pm 0.18 & 0.05\pm 0.05 & 0.13\pm 0.07\\ \cdot & \cdot & 0.53\pm 0.09 & 0.04\pm 0.05 & 0.04\pm 0.03 & 0.04\pm 0.02\\ \cdot & \cdot & \cdot & 0.29\pm 0.01 & 0.02\pm 0.01 & 0.03\pm 0.01\\ \cdot & \cdot & \cdot & \cdot & 0.31\pm 0.05 & 0.03\pm 0.02\\ \cdot & \cdot & \cdot & \cdot & \cdot & 1.31\pm 0.12 \end{array}]\text{GPa} \end{array}$$ (26)

$$\begin{array}{c} {\bar{\left[C\right]}}_{f=20\%}^{B32}=[\begin{array}{cccccc} 4.31\pm 0.14 & 1.32\pm 0.06 & 0.28\pm 0.05 & 0.02\pm 0.01 & 0.05\pm 0.04 & 0.09\pm 0.05\\ \cdot & 4.30\pm 0.12 & 0.28\pm 0.04 & 0.06\pm 0.07 & 0.03\pm 0.03 & 0.14\pm 0.26\\ \cdot & \cdot & 1.02\pm 0.05 & 0.03\pm 0.02 & 0.05\pm 0.07 & 0.04\pm 0.03\\ \cdot & \cdot & \cdot & 0.46\pm 0.05 & 0.02\pm 0.01 & 0.04\pm 0.03\\ \cdot & \cdot & \cdot & \cdot & 0.47\pm 0.01 & 0.03\pm 0.03\\ \cdot & \cdot & \cdot & \cdot & \cdot & 1.33\pm 0.02 \end{array}]\text{GPa} \end{array}$$ (27)

### Size effect and prediction of RVE size

The sample set of *V*_*k*_ and respective *n*_*k*_ in Table 6 with *k* between 1 and 6 was extracted randomly for each *f* from the network samples. *n*_*k*_ replicates the scheme found in [45]. The original publication [29] relates to the measurement error which would be rather difficult to quantify for the presented meshed fibres network systems.

**Table 6:** Sample set of random realisations for each fibre volume fraction.

Index k	1	2	3	4	5	6	Full sample	
Sub-sample size $V_{k}^{1/3}$	50	100	150	200	250	300	516	[pixel]
	0.39	0.78	1.16	1.55	1.94	2.33	4.00	[mm]
Number of realisations *n*_*k*_	140	110	80	50	30	10	2	[-]

**Fig 8:**
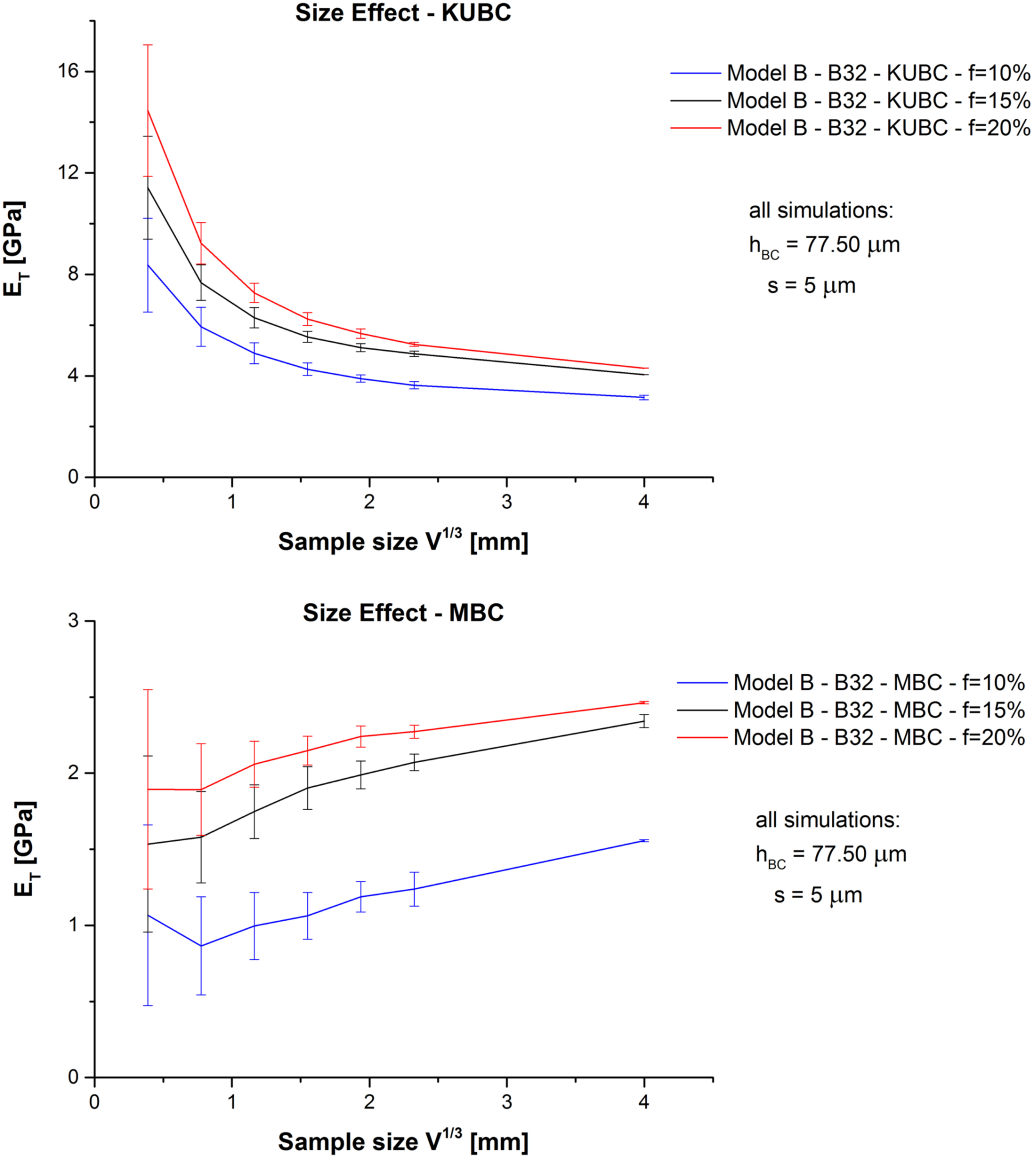
Model B—Size effect. Obtained *E*_*T*_ values of Model B depending on *V* ^1/3^ (error bar = ±*D*_*E*_*T*_(*V*^1/3^)_).

*E*_*T*_ shows for the two BC types two different size effects, decreasing for KUBC and increasing for MBC (see Fig 8). These two patterns can be found similarly in [29], there in particular for the bulk modulus under MBC, and for KUBC in [32].

The application of the regression algorithm in Eq (21) (with *Z* = *E*_*T*_) leads to the values of Table 7. Greater *f* reduces *V*_RVE,*E*_*T*__. The influence of the BC type on the material is found to be as predicted by [29]:

$$\begin{array}{c} V_{\text{RVE},\text{MBC}}>V_{\text{RVE},\text{KUBC}} \end{array}$$ (28)

**Table 7:** RVE results for KUBC and MBC, obtained by linear regression.

BC -	Values from linear regression			RVE $V_{\text{RVE},E_{T}}^{1/3}$ [mm]		
Fibre volume fraction *f*	*α* [-]	$A_{3}^{1/3}$ [mm]	*R* ^2^ [-]	*n* = 1, *ϵ*_*rel*_ = 1%	*n* = 2, *ϵ*_*rel*_ = 1%	*n* = 5, *ϵ*_*rel*_ = 1%
KUBC—10%	0.97	0.036	0.97	5.64	4.47	3.29
KUBC—15%	0.90	0.009	0.98	2.25	1.74	1.24
KUBC—20%	0.81	0.003	0.95	1.12	0.84	0.58
MBC—10%	1.41	21.274	0.89	1,210.50	1,042.56	855.84
MBC—15%	1.15	0.294	0.94	39.44	32.62	25.38
MBC—20%	0.94	0.010	0.93	5.06	3.98	2.89

For *f* = 10% under MBC, the condition of Eq (18) [29] isn’t fulfilled any more. The precision of the values for this particular simulation point could be investigated further in future work by greater sample sets.

### Prediction of fibre deformation mechanism

**Fig 9:**
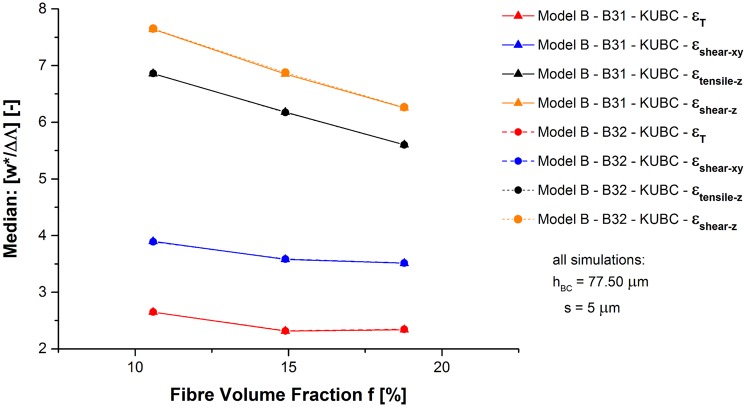
Model B—Deformation mechanism. Obtained median of [w*/ΔΛ] depending on *f*.

The fibre segment deformation inside the network sample by deflection w* (perpendicular to the fibre segment axis) is compared to the elongation ΔΛ (along the axis) by the median of their ratio under KUBC (see Fig 9). For greater simplicity, load cases *i* = 1 and 2 are summarised to *ϵ*_*T*_, *i* = 4 and 5 to the out-of-plane shear deformation *ϵ*_shear-z_. Load case *i* = 3 stands for *ϵ*_tensile-z_ and *i* = 6 for *ϵ*_shear-xy_.

Increased *f* has been shown to reduce the fibre segment length *λ* (see Table 1 and [36]) which reduces simultaneously in the present study the obtained median [w*/ΔΛ]. The obtained median ratio is in each case greater than 2, increasing for shear and out-of-plane deformation:

$$\begin{array}{c} 2<{\left[\frac{w^{*}}{\Delta \Lambda }\right]}_{\epsilon_{T}}^{\text{Median}}<{\left[\frac{w^{*}}{\Delta \Lambda }\right]}_{\epsilon_{\text{shear}-\text{xy}}}^{\text{Median}}<{\left[\frac{w^{*}}{\Delta \Lambda }\right]}_{\epsilon_{\text{tensile}-z}}^{\text{Median}}<{\left[\frac{w^{*}}{\Delta \Lambda }\right]}_{\epsilon_{\text{shear}-z}}^{\text{Median}}<8 \end{array}$$ (29)

## Conclusions and Future Work

The present study simulates the elastic mechanics of metallic fibre networks by two FE models (rigid inter-fibre joints or torsional spring connectors) and validates the results by experimentally obtained values from [36]. While [29, 32] used solid volume meshes, beam theory is shown to offer a valid simplification method for the investigated material.

BC types MBC and KUBC are simulated and a new model parameter *h*_*BC*_ is introduced for the depth to which BC are prescribed into the material along ${\underline{n}}^{in}$ on ∂*V*. MBC with fewer prescribed DOF provide a greater match to the experimental values of in-plane tensile testing than KUBC do. The results for rigid inter-fibre joints and for torsional spring connectors converge incrementally. During this process, the influence of the spring constant on the overall structural stiffness decreases. This implies for the manufacturing process that the stiffness of inter-fibre joints becomes increasingly negligible as soon as a minimum joint strength has been achieved in the sintering process.

In [29], an isotropic material behaviour was obtained for computer generated fibre networks without preferred fibre orientation direction. The material of the present study exhibits a dominantly in-plane fibre orientation [36] and an in-plane transversely isotropic mechanical behaviour. Depending on the applied BC type, a decreasing or increasing size effect is observed. Fibre segment deflection dominates the deformation inside the network over fibre segment elongation. These three findings imply for future work and future design studies that size and shape of the material are of importance, as well as the manufactured fibre orientation.

Of interest for future work is also the achievable strain magnitude in a matrix material located in the network’s void phase and the material’s suitability for mechanical bone growth stimulation [74].
